# COVID-19 ARDS: Points to Be Considered in Mechanical Ventilation and Weaning

**DOI:** 10.3390/jpm11111109

**Published:** 2021-10-28

**Authors:** Eumorfia Kondili, Demosthenes Makris, Dimitrios Georgopoulos, Nikoletta Rovina, Anastasia Kotanidou, Antonia Koutsoukou

**Affiliations:** 1 Department of Intensive Care Medicine, University Hospital of Heraklion and Medical School, University of Crete, 71110 Heraklion, Greece; georgopd@uoc.gr; 2 Critical Care Department, University Hospital of Larissa, Faculty of Medicine, University of Thessaly, 41110 Larissa, Greece; appollon7@hotmail.com; 3 1st Department of Respiratory Medicine, Medical School, “Sotiria” Chest Disease Hospital, National and Kapodistrian University of Athens, 11527 Athens, Greece; nikrovina@med.uoa.gr (N.R.); koutsoukou@yahoo.gr (A.K.); 4 1st Department of Critical Care Medicine & Pulmonary Services, School of Medicine, National & Kapodistrian “Evangelismos” Hospital, University of Athens, 10676 Athens, Greece; akotanid@med.uoa.gr

**Keywords:** COVID-19, lung injury, mechanical ventilation, weaning

## Abstract

The COVID-19 disease can cause hypoxemic respiratory failure due to ARDS, requiring invasive mechanical ventilation. Although early studies reported that COVID-19-associated ARDS has distinctive features from ARDS of other causes, recent observational studies have demonstrated that ARDS related to COVID-19 shares common clinical characteristics and respiratory system mechanics with ARDS of other origins. Therefore, mechanical ventilation in these patients should be based on strategies aiming to mitigate ventilator-induced lung injury. Assisted mechanical ventilation should be applied early in the course of mechanical ventilation by considering evaluation and minimizing factors associated with patient-inflicted lung injury. Extracorporeal membrane oxygenation should be considered in selected patients with refractory hypoxia not responding to conventional ventilation strategies. This review highlights the current and evolving practice in managing mechanically ventilated patients with ARDS related to COVID-19.

## 1. Introduction

COVID-19 may cause hypoxemic respiratory failure, meeting the criteria of acute respiratory distress syndrome [ARDS] [1,2,3,4,5,6]. The majority of patients admitted in ICU will ultimately need some form of ventilator assistance and invasive mechanical ventilation [1,4,5]. Patients undergoing invasive mechanical ventilation have significantly longer mechanical ventilation and ICU stays than those with ARDS of other causes, whereas mortality in those patients varies considerably in different reports [1,5,7]. Observational studies, including a significant number of patients with ARDS due to COVID-19, have reported similar pathophysiological and clinical characteristics and respiratory system mechanics with ARDS of other causes [1,2,3,4,5]. These findings contrast the early reports that supported the presence of distinctive phenotypes of ARDS in COVID-19 patients [8,9,10]. The management of COVID-19 patients with ARDS is challenging. Ιt has been shown that mechanical ventilation relies mainly on the strategies that have been proved protective for the lung with respect to avoiding the development of ventilator-induced lung injury (VILI) [1,2]. Furthermore, physicians may face challenges with the use of assisted mechanical ventilation in COVID-19 patients. While spontaneous efforts may help in preserving diaphragmatic strength and thereby reduce the risks associated with diaphragm atrophy and dysfunction, they may promote regional stress and strain heterogeneity, potentially having a role in the progression of lung injury [11,12].

These points have to be considered carefully in COVID-19 patients who present severe forms of ARDS and difficult weaning. This review highlights the current and evolving practice of managing mechanically ventilated patients with ARDS related to COVID-19.

## 2. Is the ARDS in COVID-19 Different from ARDS of Other Causes?

Soon after the first patients with ARDS due to COVID-19 were admitted in ICUs worldwide, the hypothesis of the existence of two distinct ARDS phenotypes was introduced: the “type L” phenotype characterized by relatively low lung elastance (high compliance), low lung weight as estimated by CT scan, and low response to PEEP, and the “type H” phenotype described by high elastance (low compliance) with extensive CT consolidations, high lung weight, and high PEEP response. Accordingly, different ventilator strategies for the patients assigned in the two different phenotypes were suggested [8,9,10].

An important issue arising from this hypothesis is whether ARDS in COVID-19 patients is a different entity from ARDS of other causes; therefore, there is a need to abandon current established ventilator strategies in favor of a new approach.

The notion that there is considerable heterogeneity in respiratory mechanics, gas exchange disarrangement, and clinical course among patients with ARDS is an essential and pervasive finding [13,14]. Variations in lung compliance are not a specific feature of mechanically ventilated patients with COVID-19. Evidence from large ARDS trials supports that lung compliance in ARDS patients varies across a broad spectrum. Interestingly, a quarter of patients randomized in the ARDS-NET study had plateaued pressures in the range of 10 to 20 cmH_2_O, which is consistent with high lung compliance [15].

Subsequent cohorts included a substantial number of mechanically ventilated patients with COVID-19 and reported similar clinical characteristics and respiratory system mechanics with those reported in prior large cohorts of patients with classical ARDS [1,2,3,4,16]. Moreover, it has been shown that patients with ARDS due to COVID-19 highly respond to established ventilation strategies, corresponding to patients with ARDS of other causes [16,17,18,19]. With respect to lung recruitabilty in patient ARDS due to COVID-19, two small observational studies reported opposite results that are probably related to the timing of recruitability assessment in the course of the disease [16,18]. Plausibly in some patients, the reported high respiratory compliance may be related to the timing of the initiation of mechanical ventilation. Specifically, in COVID-19 patients, intubation time varies considerably depending on the criteria set institutionally and the available resources [20]. It follows that patients who are intubated prematurely in the early phase of COVID-19 disease may present higher respiratory system compliance than those who have delayed or deferred commencement of mechanical ventilation [20].

Therefore, although some patients may exhibit high compliance, at least early in the course of the disease, there are insufficient patient data and certainly no experimental evidence to support the proposed model of different phenotypes.

## 3. How to Ventilate a Patient with ARDS due to COVID-19

Since ARDSs due to COVID-19 have similar pathophysiological, clinical characteristics, and respiratory system mechanics with ARDS of other causes, mechanical ventilation in those patients should rely on established protective ventilator strategies that target at minimizing ventilator-induced lung injury (VILI) [21,22,23,24].

### 3.1. Controlled Mechanical Ventilation

#### 3.1.1. Ventilator Strategies

Conventionally, controlled mechanical ventilation is applied in the early stage of ARDS, and volume control remains the most common ventilation mode during the first days of mechanical ventilation [25]. Protective mechanical ventilation during controlled modes aims to minimize stress and strain, the main determinants of VILI [22,24]. Table 1 summarizes the main ventilator and non-ventilatory strategies.

#### 3.1.2. Setting FiO_2_

The administration of supplementary O_2_ therapy in mechanically ventilated patients with ARDS aims to improve oxygenation, albeit the optimal oxygenation target remains controversial [26,27]. The strategy of permissive hypoxia defined as SaO_2_-82–88% had previously been proposed in patients with severe ARDS in order to minimize the potential of hyperoxia-induced effects [28].

However, a retrospective study reported that patients with low PO_2_ (<72 mmHg) have a significantly higher incidence of long-term cognitive dysfunction than those with higher PO_2_ [29]. Two recent randomized studies investigated the effect of conservative versus liberal oxygen therapy in mechanically ventilated ARDS patients, and the studies yielded controversial results [30,31]. In the absence of conclusive studies and based on the harmful effects of hypoxia (tissue hypoxia) and hyperoxia (increased formation of reactive oxygen species, ROS), exposure to both hypoxia and hyperoxia should be avoided [32]. Currently, the standard recommendations of care in patients with ARDS suggest a target for conservative arterial oxygenation (PaO_2_ = 65–75 mmHg; SaO_2_ = 90–95%) [33].

#### 3.1.3. Setting Tidal Volume

Based on an evidence-based approach, setting tidal volume (Vt) in patients with ARDS due to COVID-19 should rely on established protective ventilation strategies. Since the results of the ARDSnet study, setting low tidal volume <6 mL/Kg of predicted body weight (PBW) and maintaining end-inspiratory pressure (Pplat) <30 cmH_2_O represent the fundamentals principles of protective mechanical ventilation in patients with ARDS [15,34].

However, PBW is poorly related to resting lung volume, and a similar tidal volume may cause different strains and stresses (the main determinants of VILI) in patients with the same anthropometric characteristics [35].

The ratio of Vt to respiratory system compliance (Crs), termed driving pressure (ΔP, calculated as the difference between Pplat and PEEP), better reflects lung stress since respiratory system compliance is strongly related to functional lung size. Setting Vt to achieve a ΔP < 14 cmH_2_O has been independently associated with lower mortality in ARDS patients [36,37]. Similar results were reported recently in COVID-19 patients [20].

Another method for titration tidal volume and other ventilator settings to minimize VILI could be targeting a certain level of mechanical power (MP), which describes the total energy applied to the respiratory system in the unit of time. The concept of MP has been introduced by Gattinoni a few years ago for estimating the contribution of various ventilator settings on the development of VILI, e.g., VT, respiratory rate, flow, and airway pressures [38].

Experimental and clinical studies have shown that an increase in MP above a certain level is associated with pulmonary edema formation and is independently associated with mortality [39,40,41,42,43].

A recent study based on retrospective analysis of previous studies in patients with ARDS has shown that the combination of driving pressure and the respiratory rate (4*Dp*RR) is associated with increased mortality and may serve as a simplified surrogate of MP [44].

In patients with suspected high chest wall elastance, estimation of the end-inspiratory transpulmonary pressure (PL, the difference between Pplat and oesophageal pressure (Poes) at end-inspiration) may be required [45]. Although established targets for safe limits of end-inspiratory PL are lacking, keeping it below 18–20 cmH_2_O has been widely considered a safe range to prevent regional lung overdistension [46].

A research group has suggested a liberal Vt approach (8–9 mL/kgPBW) in patients with ARDS due to COVID-19 who initially exhibit nearly normal respiratory system compliance for avoiding excessive hypercapnia attributed to abnormal vascular components of dead space [8,9,10]. However, patients with COVID-19 commonly present excessive inflammatory responses that may predispose them toward ventilatory-induced lung injury, resulting in rapid deterioration in respiratory system compliance. Hence, setting a Vt higher than that suggested by lung-protective ventilatory strategy could conceivably increase stress and strain and can induce adverse impacts on patients with initially normal respiratory system mechanics [22,24].

In clinical practice, as an initial approach, a tidal volume of ≤6 mL/kg PBW should be applied and subsequently adjusted, targeting Pplat, ΔP, and end-inspiratory PL values within the range described above. Low Vt may cause hypercapnia and concomitant respiratory acidosis. In order to limit hypercapnia, minute ventilation can be increased by setting the breathing frequency up to 30/min. Measures should be considered for decreasing instrumental dead space, such as replacing heat and moisture exchange filters with a heater humidifier [47]. Although a safe lower limit of pH is unknown, a pH up to 7.22 is considered to have minimal deleterious effects in all patients except those with elevated intracranial pressure or pulmonary hypertension.

#### 3.1.4. Setting PEEP

Applying PEEP in patients with ARDS represents one of the fundamentals of protective mechanical ventilation. The application of PEEP mitigates the risk of atelectotrauma by preventing the cycling opening and closing of unstable alveoli and improving lung compliance and homogeneity by increasing the number of open alveoli [46].

Patients with ARDS respond varyingly to an increase in PEEP, presenting high or low lung recruitability [48]. Applying a high PEEP level in the first group will reduce mechanical strain and atelectotrauma and decrease dead space ventilation and shunt. However, in patients with low lung recruitability (i.e., those with primary focal lung injury), a high PEEP level will cause additional distension of already aerated alveoli and increase dead space ventilation. Therefore, before setting the adequate PEEP, lung recruitability should be assessed.

Different methods have been proposed for assessing lung recruitability in patients with ARDS. In clinical practice, the most common method for assessing lung recruitability and titrating PEEP is by applying different levels of PEEP and monitoring oxygenation, respiratory system compliance, the end-inspiratory pressure (Pplat), and the driving pressure (ΔP). In highly recruitable lungs, increasing PEEP is associated with improved oxygenation, respiratory system compliance, and decreased ΔP. CT scan remains the most accurate method of response to different levels of PEEP [49]. Nevertheless, the method is time-consuming and complicated as it requires patient transportation out of the ICU environment, and in patients with COVID-19, this carries an increased risk of contamination. An alternative method for assessing lung recruitability and titrating PEEP at the bedside is electrical impedance tomography (EIT), a non-invasive radiation-free imaging method; however, its availability is currently limited [50,51].

A simple bedside method for assessing lung recruitability has been recently proposed: the recruitment to inflation (R:I) ratio [18,52]. R:I is derived during a one-breath maneuver in which PEEP decreases by 10 cmH_2_O and can be easily calculated with an online application (rtmaven.comaccessed on 10/10 2021). Values > 0.5 indicate potential lung recruitability.

PEEP titration based on end-expiratory transpulmonary pressure (Plendexp) may be required in patients with severe ARDS and low chest wall compliance [45]. Due to reduced chest wall compliance, edema, or abdominal distension, oesophageal pressure (Poes) may be elevated, and the calculated transpulmonary pressure (the difference between Paw and Peos) can be negative at end-expiration. Negative PLendexp indicates closed or compressed airways or atelectatic lungs. Thus, PEEP could be increased until transpulmonary pressure becomes positive at end-expiration to keep the airways open. Increasing the PEEP aiming to Plend-exp ≥0 cmH_2_O has been shown to improve oxygenation compared to titration based on the table in the ARDSNetwork study [45].

Since ARDS in COVID-19 presents similar characteristics and respiratory mechanics with the ARDS of other causes, it is not unreasonable to rely on strategies already established in patients with ARDS of other causes when setting PEEP in those patients. Recent observational studies in patients with ARDS due to COVID-19 report that the median PEEP level was ≥10 cmH_2_O, which is moderately higher than that reported in the LUNG SAFE study [2,7]. [8.4 cmH_2_O]. The higher level of PEEP in COVID-19 patients may be attributed to the higher incidence of severe hypoxemia; baseline PaO_2_/FiO2 is frequently <150 mmHg at the commencement of mechanical ventilation [20]. Conventionally, an initial PEEP > 8 cmH_2_O is selected in moderate to severe ARDS, and further titration is based on the indices described above byconsidering lung recruitability and the potential effects of PEEP on systemic and pulmonary circulation.

#### 3.1.5. Recruitment Maneuvers

A recruitment maneuver (RM) is the intentional application of elevated transpulmonary pressure to reopen previously collapsed lung units, thus increasing the lung units available for gas exchange. RM should be considered before any change in the level of applied PEEP or in the case of intentional or accidental ventilator circuit discontinuation [53,54]. Different techniques of RM have been proposed [53]. As sufficient evidence for distinguishing which technique is superior is lacking, in everyday clinical practice, the choice of the technique for performing RM is determined by individual bias. Several studies have been shown the beneficial effects of RM on the improvement of oxygenation in patients with ARDS [55,56]. It should be observed that the applying RM may be associated with hemodynamical compromise, whereas other more serious adverse events, such as barotrauma, infrequently occur [55,56,57,58].

#### 3.1.6. Adjuvant Non-Ventilatory Strategies

##### Prone Positioning

Prone positioning has been proven to be one of the most effective interventions in patients with moderate to severe ARDS and is associated with increased survival [59].

A multicentre randomized controlled trial found that placing patients with severe ARDS (PaO_2_/FiO_2_ < 150 mmHg) in the prone position for at least 16 h/day improved survival compared to the semi-recumbent supine position [59]. The beneficial effects of prone positioning in ARDS patients are attributed to lung ventilation and lung perfusion alterations. Normally, ventilation favors ventral lung regions, and pulmonary perfusion preferentially distributes to the dorsal lung regions [60]. In patients with ARDS, lung edema further diminishes dorsal regions aeration, resulting in shunt aggravation and increased low V’/Q’ in these regions. Prone position increases the aeration of dorsal lung units, and as pulmonary perfusion remains preferentially distributed to the dorsal lung regions, ventilation/perfusion matching improves and shunt fraction decreases, causing an increase in PaO_2_ and a decrease in PaCO_2_ in several patients [60,61]. Furthermore, recent data support that prone positions increase lung homogeneity, especially at high PEEP level [60,61].

Moreover, the more homogenous lung ventilation and increased size of the aerated lung may protect the lung from abnormal stress and strain and mitigate the risk of VILI [62,63]. Recent clinical trials suggest that prone positioning sessions should be initiated early (within two days on mechanical ventilation) in patients with moderate to severe ARDS (PaO_2_/FiO_2_ ratio <200 mmHg) [23,59].

Prone positioning has been widely used in mechanically ventilated patients with ARDS due to COVID-19 as part of ventilation management in patients with refractory hypoxia. A scoping review reports that the rate of prone positioning in patients with COVID-19 ranges from 17 to 81% [2], which is significantly higher than the rate of 7.9% reported in the LUNG SAFE study [7]. A bicentric observational retrospective study reported significant improvement of oxygenation on prone positioning in 77% of proning sessions, although 14% of the cases showed slow response in oxygenation improvement (after 9.5 h) [64].

A recent observational study reported that in patients with ARDS due to COVID-19, prone positioning for a prolonged period [36 h] was safe and associated with more pronounced impacts on oxygenation than 16 h of prone positioning [65]. Accordingly, prone positioning should be applied for extended periods in the absence of contraindications or significant side effects. In the condition of work overload for healthcare assistants, this strategy might reduce the number of pronation cycles required for a single patient.

Optimal timing and criteria for discontinuing prone ventilation are somewhat unclear and should be evaluated individually. It is not unreasonable to implement criteria similar to those in studies that have shown benefits in non-COVID-19 related ARDS: PaO_2_/FiO_2_ ≥ 150 mmHg, FiO_2_ ≤ 0.6, and PEEP ≤ 10 cmH_2_O maintained for at least four hours after the end of the last prone session [23].

Prone position in mechanically ventilated COVID-19 patients has been associated with a favorable outcome. A recent observational study reported lower in-hospital mortality in mechanically ventilated patients treated with early proning than compared to patients whose treatment did not include early proning [66].

It should be emphasized that prone positioning sessions should be combined with other lung-protective strategies (low VT, low Pplat and driving pressures, and individualized PEEP titration).

##### Neuromuscular Agents

The beneficial effect of neuromuscular blockers (NMBA) on the outcome of mechanically ventilated patients with ARDS remains controversial since two large randomized trials reported inconsistent results [67,68]. However, NMBA should be considered in specific groups of ARDS patients as it may be associated with a favorable outcome. The exact mechanism is unidentified, and it is hypothesized that lower transpulmonary pressure and improved patient–ventilator interaction facilitated lung-protective ventilation. Current guidelines suggest utilizing NMBA in patients with ARDS for no longer than 48 h and utilizing significant patient–ventilator interaction (i.e., reverse triggering) that restrains lung-protective ventilation in patients with severe hypoxemia and those with markedly high respiratory drive despite deep sedation [23,69].

NMBA has been used widely in mechanically ventilated COVID-19 patients. Recent observational studies reported that the rate of NMBA use in those patients ranges from 22–88% [1,2,70], significantly higher than that reported in previous studies in patients with ARDS of other causes [7]. Furthermore, in about two-thirds of patients, the duration of NMBA is longer (median five days) than the recommended two days [2]. The frequent and prolonged prone positioning along with high respiratory drive that these patients frequently exhibit may explain both the high rate and the longer duration of use of NMBA in COVID-19 patients.

The judicious use of NMBA in mechanically ventilated COVID-19 patients is suggested in patients with remarkably high respiratory drive, even when on deep sedation. NMBA in those patients facilitates lung-protective ventilation and, by preventing the strong inspiratory efforts, may improve patient–ventilator synchrony and decrease the risk of self-inflicted lung injury.

### 3.2. Assisted Mechanical Ventilation

MV discontinuation requires reduction in mechanical ventilatory support and placement of work of breathing (WOB) on the respiratory muscles [71,72,73]. When respiratory failure due to COVID-19 ARDS recedes and the patient’s condition improves, the patient may be allowed to resume spontaneous breathing and assisted mechanical ventilation (AMV) should be considered [71,72,73]. The decision/criteria for switching to AMV are subjects of considerable discussion. Any strategy has to assess whether the patient is deemed ready for AMV. The current recommendations for non-COVID patients could also be applied in COVID-19 patients, emphasizing that a conservative strategy may be associated with improved outcomes [74,75,76,77]. Solid evidence-based recommendations for ventilation strategies during AMV in patients with COVID-19 ARDS are lacking [76,77]. It has been proposed that clinicians should follow the recommended principles for non-COVID ARDS (a protective strategy based on low Vt and airway pressures) [75,78]. Both conventional (pressure support, PS) and proportional modes (proportional assist ventilation with adjustable gain factors; PAV+, and neurally adjusted ventilatory assist; NAVA) can be used [25,79,80,81,82,83,84]. Although studies are lacking in COVID-19 patients, proportional modes over PS are preferable as these modes follow patients’ ventilation demands and are associated with better patient–ventilator synchrony [79,81,83,84]. In addition, PAV+ automatically calculates respiratory system mechanics (compliance and resistance) and drives pressure, a tool that may assist the decision-making process [81].

#### Specific Points to Be Considered in Assisted Mechanical Ventilation

##### The Role of Spontaneous Breathing

Assisted mechanical ventilation (AMV) should be considered as earlier as possible in the course of mechanical ventilation as spontaneous breathing may improve lung ventilation/perfusion matching and shunt by recruiting dorsal atelectatic regions, prevent ventilation-induced diaphragmatic dysfunction (VIDD), and reduce sedation requirements [72,85,86,87]. Nevertheless, spontaneous breathing may per se worsen lung injury resulting in so-called patient inflicted lung injury (P-SILI) [11,12,73,88,89,90].

Different mechanisms have been proposed for the pathogenesis of P-SILI. Vigorous spontaneous breathing decreases pleural pressure and increases transpulmonary pressures and tidal volumes, exacerbating lung stress and strain for the same lung mechanics. Moreover, inspiratory effort produces a more negative pleural pressure in the dependent lung regions than compared to non-dependent lung regions in the case of lung injury. As a result, air is moving from non-dependent to dependent areas early in inspiration, causing significant distention of the dependent areas and lung injury aggravation, a phenomenon described as pendelluft. In addition, patient–ventilator asynchrony mainly doubling triggering and breath stacking may further augment transpulmonary pressures and tidal volumes despite lung-protective strategies. Recent experimental findings suggested that effort-dependent lung injury was minimized by high PEEP in severe ARDS; however, more data, especially from clinical studies, are necessary to provide insight for the optimum ventilatory strategy in this setting [91,92].

##### Transpulmonary Pressures

Spontaneous breathing during MV in injured lungs needs careful attendance because spontaneous efforts may promote regional strain and strain heterogeneity and may have a role in the progression of lung injury [77,88,93]. The regional volumetric deformation of the lung during high strain-spontaneous breathing and the transduction of mechanical power delivered to the lung may result in a type of effort-induced lung injury [89,90,94]. Monitoring transpulmonary pressure may be required during assisted mechanical ventilation as it may serve as an indicator of the inspiratory efforts’ intensity [95,96,97,98]. Low swing pressures (both esophageal and transdiaphragmatic <7 cmH_2_O) were associated with low inspiratory efforts in terms of the pressure-time product (PTPPdi/min <50 cmH_2_O.s/min), whereas relatively high efforts (PTPPdi/min >150 cmH_2_O.s/min) were associated with increased swings (14–18 cmH_2_O) [98]. Therefore, clinicians should be vigilant if vigorous spontaneous efforts are present during weaning.

##### Inspiratory Respiratory Drive

Increased respiratory drive may be deleterious as it may promote dynamic hyperinflation and increased oxygen consumption [97,99]. High respiratory drive may be associated with patient self-inflicted lung injury [P-SILI], and this may be a pathophysiologic mechanism in COVID-19 ARDS for the progression of the syndrome (from type L to type H phenotype as previously suggested) [8,100]. In addition, increased WOB due to high ventilation demands (pneumonia, systemic inflammation, uncontrolled delirium, presence of superinfection, drug withdrawal reaction, and acidosis) is associated with weaning failure and prolonged MV duration [76,77,101,102]. Therefore, it is essential to prevent and control these conditions during weaning, and respiratory drive must be monitored and managed optimally [101,102]. Although specific indices for direct respiratory drive evaluation are lacking, monitoring variables deemed surrogates for the respiratory drive, such as the airway occlusion pressure (P0.1) or the swing in airway pressure generated by respiratory muscle effort when the airway is briefly occluded (Δ*P*occ), should be considered alternatively [103,104]. A recent study evaluated airway occlusion pressure [P0.1] in mechanically ventilated COVID-19 patients and showed that P0.1 above 4 cm H_2_O could predict the worsening of respiratory failure in the next 24 h [78,103]. On the other hand, a recent study showed that measuring Δ*P*occ may enable accurate non-invasive detection of elevated respiratory muscle pressure and transpulmonary driving pressure [104].

##### Patient–Ventilator Asynchrony

Patient–ventilator asynchrony is associated with adverse outcomes and has been identified as a risk factor for unsuccessful weaning [105,106]. Patient–ventilator asynchrony may occur during AMV due to uncoupling between the patient and the ventilator in terms of timing (matching between neural and mechanical time) and ventilatory assistance [107,108]. Ineffective efforts, autotriggering, and double triggering are the most common forms of asynchrony [82,109,110,111]. Ineffective efforts and breath stacking may be frequently encountered in COVID-19 patients due to myopathy and increased respiratory drive, respectively.

Ineffective efforts may occur during either the inspiratory or the expiratory ventilator phase. Their incidence depends on several factors related to the patient’s medical condition, ventilator settings, and sedation depth. It usually implies a decrease in inspiratory muscle pressure (i.e., sedation, sleep, respiratory or metabolic alkalosis, and polyneuromyopathy) or factors promoting dynamic hyperinflation (i.e., obstructive lung disease, tachypnea, high tidal volume due to high level of assist, and delayed cycling off). Double triggering (or breath stacking) may occur when the patient’s demand is high, and his inspiratory effort continues throughout the preset ventilator inspiratory time and remains present even after the end of ventilator inspiratory time [34]. This phenomenon may result in the delivery of two consecutive breaths for one patient’s effort. Short inspiratory times, usually accompanied by low tidal volume and high respiratory drive, are the cause for double triggering.

##### Airway Pressures

Plateau end-inspiratory airway pressure (Pplat) may help estimate respiratory system compliance and driving pressure—both are associated with major outcomes in ARDS [36,112,113]. Recent investigations showed that high driving pressures and low compliance during AMV are also associated with adverse outcomes [114]. Despite the great interest in implementing the measurement of Pplat during AMV, there are limitations in the accuracy of the measurement that are mainly due to mismatch between the end of neural inspiration and end of mechanical inflation ^113^. Undoubtedly, high driving pressures in AMV are a concern and require further investigation; it may result from expiratory impaired lung mechanics and/or decreased chest wall compliance [114,115]. Therefore, it seems prudent to implement further diagnostic workup (i.e., measurement of esophageal pressures). The most important points to consider during assisted mechanical ventilation are summarized in Table 2.

## 4. Weaning from Mechanical Ventilation

Weaning from MV covers the entire process of liberating the patient from mechanical support and the endotracheal tube. There are different stages in the weaning process, including the treatment of acute respiratory failure, assessment of readiness to wean, the performance of a spontaneous-breathing-trial (SBT), and finally extubation [116]. The time spent in the weaning process is estimated at 40–50% of the total duration of mechanical ventilation, and delayed or prolonged weaning may expose the patient to an increased risk of complications [74,117]. Patients with COVID-19 commonly exhibit difficult or prolonged weaning. Recent USA and European cohorts reported that about half of these patients remained intubated for almost 14 days [118,119]. Additionally, patients who were older (>65 years) and/or obese (BM1 > 30) were at higher risks for difficult weaning [118,119]. Pathophysiology of weaning failure is multifactorial; the pathophysiologic factors associated with weaning failure are summarized in Table 3. Respiratory pump insufficiency results from a decrease in respiratory neuromuscular capability and/or increased respiratory muscle workload; it is probably the most common cause of weaning failure in COVID-19. Inspiratory muscle dysfunction is frequently accounted for in COVID-19 patients, especially in those with prolonged mechanical ventilation and/or those treated with muscle relaxants for prolonged periods. Increased respiratory muscle load is mainly attributed to reduced respiratory system compliance due to either COVID-19 or pneumonia, pulmonary edema, fibrosis, and hemorrhage [120,121,122]. Several clinical, physiologic and non-invasive ultrasound indices are currently available for identifying patients who will have successful weaning [116,123]. 

Moreover, different tools have been addressed to prevent and treat post-extubation respiratory failure. The use of pre-emptive noninvasive ventilation (NIV) may be beneficial as a bridge to full spontaneous breathing in hypercapnic patients and in selected hypoxemic patients at high risks of weaning failure [124,125]. High flow nasal cannula (HFNC) oxygen therapy is an additional optimal tool for hypoxemic patients, and it is easily tolerated by the patients and may reduce the need for reintubation [126,127].

## 5. When Should We Consider Referring a Patient for Extracorporeal Membrane Oxygenation

The use of extracorporeal membrane oxygenation (ECMO) for ARDS has been previously reported during the 2009 influenza A (H1N1) pandemic, as well as the Middle East respiratory syndrome coronavirus (MERS-CoV) outbreak [128,129,130]. ECMO is used in patients with ARDS due to COVID-19 [129,131,132]. Although early reports on ECMO use for COVID-19-related ARDS were disappointing, raising concerns regarding ECMO use in this patient population [131,132,133,134,135,136], later data reported considerably better outcomes [131,132,135,136]. Differences in patients’ clinical characteristics and resource management, along with organizational factors, may be responsible for the variations reported in the outcomes of different studies. Several organizations (World Health Organization, WHO; Extracorporeal Life Support Organization, ELSO; the Surviving Sepsis Campaign, SSC) recommend the consideration of veno-venous ECMO (V-V ECMO) in selected patients with severe ARDS and refractory hypoxaemia for conventional care (appropriate PEEP, neuromuscular blockade, prone position, and optimal ventilation) and referral in specialized ECMO centers [78,137,138]. V-V ECMO alleviates the respiratory system by improving oxygenation and by providing the benefit of lung protective ventilation. Veno-arterial (V-A) ECMO is recommended for patients with circulatory disorders, specifically in those with evidence of refractory left ventricular dysfunction. Although ECMO in several cases provides good therapeutic effects in COVID-19 patients, it can also interfere with normal physiology and induce immune and coagulation abnormalities [138,139]. Several mechanical complications, such as oxygenator dysfunction, pump failure, circuit obstruction, and cannula dysfunction, are reported in 28% of patients on ECMO by the ELSO Registry of ECMO in COVID-19 [132].

It is acknowledged that ECMO induces a pro-coagulant effect. Coagulation factors, as time passes, are eliminated due to their binding to the ECMO surface coating material. Therefore, a high incidence of venous thrombosis [33%] and pulmonary embolism [29%] is reported in COVID-19 patients receiving ECMO, whereas severe cerebral bleeding or severe bleeding is relatively rarely reported [138,140]. ECMO may also modulate inflammatory activation by reducing systemic inflammatory mediators through protective ventilation and the establishment of normoxia. Therefore, the timely use of ECMO is crucial for reducing systemic inflammation induced by severe hypoxia [141].

Indications and contraindications for the use of ECMO are shown in Table 4. In general, previously healthy young patients with single organ failure should have a priority for ECMO since they are more likely to receive the maximum benefit [131,142]. In a recent study on patients with severe refractory hypoxemic respiratory failure, V-V ECMO reduced mortality from 65 to 45% compared to conventional ventilation in a selected subpopulation (ECMO within seven days after intubation; young patients with no severe comorbidities) [143].

## 6. Conclusions

COVID-19 may cause hypoxemic respiratory failure and acute respiratory distress syndrome [ARDS]. Recent data have shown that ARDS related to COVID-19 shares common pathophysiological and clinical features with ARDS of other causes. Therefore, the aim to mitigate ventilator-induced lung injury is mandatory, and protective mechanical ventilation should be pursued in COVID-19 patients with ARDS. Assisted mechanical ventilation should be considered as early as possible in the course of mechanical ventilation; however, increased respiratory drive, vigorous spontaneous breathing, and patient–ventilator asynchrony should be carefully evaluated since they may be associated with patient self-inflicted lung injury. ECMO should be a therapeutic intervention in selected patients with severe ARDS and refractory hypoxemia to optimal ventilation, and these patients should be referred to specialized centers.

## Figures and Tables

**Table 1:** Ventilator and non-ventilator strategies during controlled mechanical ventilation.

STRATEGY	TARGET	RATIONALE-CONSIDERATIONS	Quality of Evidence
Ventilator strategies			
FiO_2_	SaO_2_	To avoid complications related to either hyperoxia or hypoxia	Controversial
Tidal volume	≤6 mL/kg/PBW	Low tidal volume improves outcome in patients with ARDS.	High
	Pplat < 30 cmH_2_O	Pplat as a surrogate of stress	Low
	PL at end-inspiration < 18–20 cmH_2_O	The stress in the lungs at a given lung volume. Consider in patients with suspected high chest wall elastance	Low
	ΔP < 14 cmH_2_O	Individualizes VT to functional lung size (Crs). The strongest predictor of mortality in recent studies.	High
PEEP	Individualizedbased on assessment of lung recruitability	Improves inhomogeneity by recruiting closed alveoli and preventing cyclic collapseConsider higher PEEP in patients with high lung recruitability	High
	PL at end -expiration > 0 cmH_2_O	Considered in patients with suspected high pleural pressure	High
Non-ventilator strategies			
Prone position	Up to 36 h/sessions	Increases lung homogeneity and size of aerated lung; improves V/Q inaqualities and decrease shuntConsider proning early in the course of mechanical ventilation in patients with moderate to severe ARDS	High
Neuromuscular blockade	<48 h infusion (Cisatracurium)	Considered in patients with severe hypoxemia, significant patient–ventilator asynchrony that restrains lung-protective ventilation, and in patients with markedly high respiratory drive despite deep sedation	Controversial

PBW = predicted body weight; Pplat = end-inspiratory pressure; PL = Transpulmonary pressure; ΔP = Driving pressure; *Crs* = Respiratory system compliance.

**Table 2:** Important points to consider during assisted mechanical ventilation.

**Vigorous spontaneous efforts during AMV** (may increase lung stress/and strain) Assess inspiratory efforts by monitoring transpulmonary swing pressures
**Increased respiratory drive** may be deleterious Assess P0.1, ΔPocc
**Patient–Ventilator Asynchrony** (most commonly with increased respiratory drive, excessive sedation, and hyperinflation) Assess especially for ineffective efforts, autotriggering, and double triggering (or other types of asynchrony)
**Airway Pressures** (are associated with major outcomes in ARDS) Assess Driving Pressure and Ppl
**Vigorous spontaneous efforts during AMV** may increase lung stress/and strain Asses inspiratory efforts by monitoring transpulmonary swing pressures

AMV = Assisted Mechanical Ventilation; P0.1 = airway occlusion pressure; ΔPOcc = airway pressure swing generated by respiratory muscle effort when the airway is briefly occluded; ARDS = Acute Respiratory Disease Syndrome; Ppl = Plateau airway pressure.

**Table 3:** Pathophysiologic factors associated with weaning failure.

**Neurological factors**	Decreased respiratory center output, electrolyte disorders, sedation, and sleep apnea
	Phrenic nerve dysfunction
**Muscle pump dysfunction**	Hyperinflation, acidosis-electrolyte disorders, malnutrition, and critical ilness neuromyopathy Diaphragmatic dysfunction
**Increased ventilation demands**	Increased CO_2_ production Dead-space ventilation
**Increased mechanical load**	Increased airway resistance (tube, central, or smaller airways)
	Reduced lung compliance (alveolar filling, atelectasis, and fibrosis)
	Increased chest wall elastance
	Reduced lung elastic recoil (COPD)
	Intrinsic PEEP
**Cardiovascular**	Increased metabolic demand Increased venous return Increased left ventricular afterload
**Endocrine dysfunction**	Hypothyroidism and myxedema
**Psychological factors**	Anxiety, delirium, and sleep deprivation

**Table 4:** Indications and contraindications for ECMO use in COVID-19 patients.

**Indications**
Refractory hypoxemia and worsening hypercapnia despite optimal ventilation strategies (neuromuscular blockade, prone positioning, high PEEP, and inhaled nitric oxide) Mechanical ventilation <7 days Should be considered when risk of death is estimated to be greater than 50% and start when it reaches or exceeds 80% Severe air leak syndrome Complicated with severe myocarditis or cardiogenic shock
**Contraindications**
*Absolute* Significant or multiple comorbidities that cannot be recovered Severe immunosuppression Sepsis and bacteremia Contraindications to systemic anticoagulation Severe multiple organ failure Severe aortic dissection Acute intracerebral hemorrhage Irreversible severe brain damage Critical congenital heart disease Chronic lung disease/uncontrolled metastatic disease Lethal chromosomal anomalies (e.g., trisomy 13 or 18) *Relative* Age ≥65 years Obesity (BMI > 30) Prolonged ventilatory support Frailty Allosensitization with prolonged waitlist time Limitations in vascular access

## Data Availability

Not applicable.
